# Total Thyroidectomy by Median Sternotomy for Treatment of Substernal Goiter: A Case Report

**DOI:** 10.7759/cureus.51967

**Published:** 2024-01-09

**Authors:** Henrique C Ferreira, Carolina P Diniz, Bernardo R Guimarães, João Bosco L Camargo Neto, Guilherme C Rezende

**Affiliations:** 1 Medicine and Surgery, Universidade de Brasília (UnB), Brasília, BRA; 2 Thoracic Surgery, Universidade de Brasília (UnB), Brasília, BRA

**Keywords:** thyroid, hyperthyroidism, anterior mediastinal mass, partial sternotomy, substernal goiter

## Abstract

Substernal goiter is a rare presentation of goiter but relatively frequent cause of anterior mediastinal mass. Symptomatic patients should be treated surgically with a total or partial thyroidectomy via a cervical or thoracic approach. This case report of a woman with a large symptomatic substernal goiter illustrates how the option to perform a partial median sternotomy may be useful when attempting a cervical approach. This allows for better visualization and resection of large masses and minimizes the risk of complications such as recurrent laryngeal nerve injury. Furthermore, it reinforces that cases of substernal goiter should be treated at specialized centers in order to minimize complications and reach better patient outcomes.

## Introduction

Substernal goiter consists in thyroid tissue within the thoracic cavity due to an enlarging thyroid gland. This rare presentation of goiter makes up 1% of total goiter cases and 7% of mediastinal masses [1]. It usually affects women above 60 years old and though it is a benign disease, its proximity to the great vessels and recurrent laryngeal nerve may cause concern due to the risk of compression of these structures [2].

Goiter is often associated with hyperthyroidism, which prompts surgical treatment. Thyroidectomy is mainly performed through a transcervical approach but may require a transsternal approach in 1 to 7.6% of cases [2]. The choice between the transcervical and transsternal approach should take into consideration the size of the mass as well as its location and careful analysis of imaging exams. This case underscores the importance of the appropriate surgical route when treating patients with a large goiter.

This article was previously presented as a meeting abstract at XXII Congresso da Sociedade Brasileira de Cirurgia Torácica on May 14, 2021.

## Case presentation

A 61-year-old woman presented with the chief complaint of retrosternal burning pain and palpitations. On further questioning she also reported insomnia, anorexia, and weight loss of 3 Kg over the last three months. Her past medical history included type 2 diabetes mellitus and hypertension, both diagnosed four years before presentation and well managed. On physical examination the patient was tachycardic, diaphoretic, and had a large mass in the anterior cervical region on the topography of the thyroid gland.

A chest X-ray showed an extensive mass in the anterior mediastinum. A computed tomography of the chest showed a large, expansive, solid, heterogeneous lesion occupying the anterior mediastinum and reaching the right atrium, measuring 16.0 x 10.7 x 7.3 cm, with an estimated volume of 650 cm^2^, extending from C7 to T7, and suggestive of substernal goiter.

**Figure 1:**
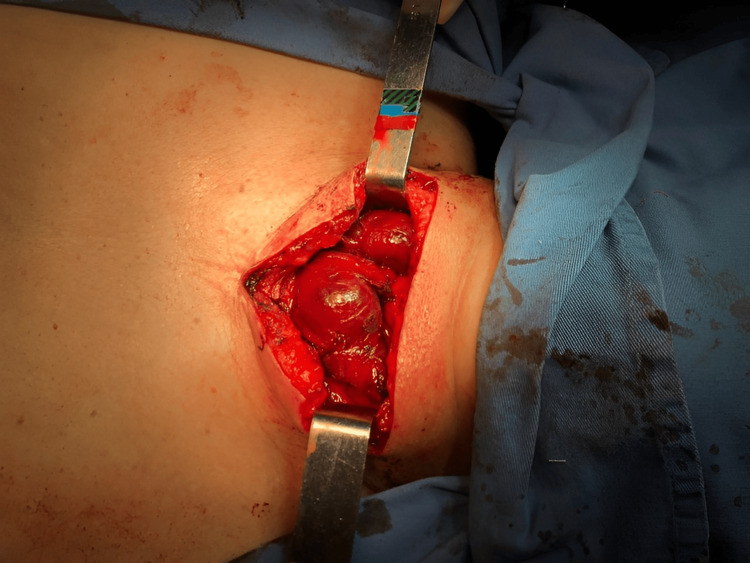
Cervicotomy with medial partial sternotomy visualizing diffuse nodular goiter.

The patient was referred and evaluated by the thoracic surgery team, on examination she had a painless enlarged thyroid gland with fibroelastic consistency and no audible murmurs. She was diagnosed with a substernal goiter and underwent elective total thyroidectomy by partial median sternotomy. The surgery began with a median cervical approach, revealing diffuse nodules and inferior protrusion to the mediastinum (Figure 1).

**Figure 2:**
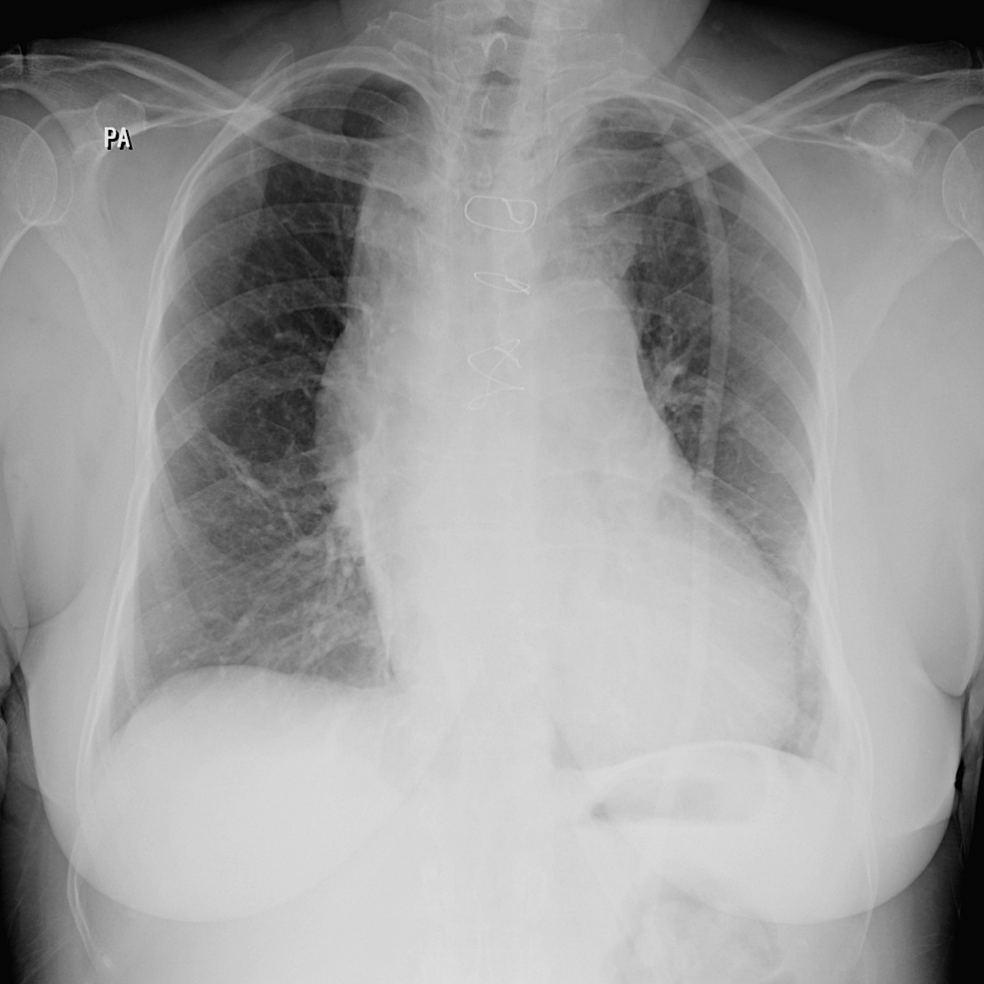
Post-operative chest X-ray shows partial median sternotomy closed with stainless steel wire sutures.

**Figure 3:**
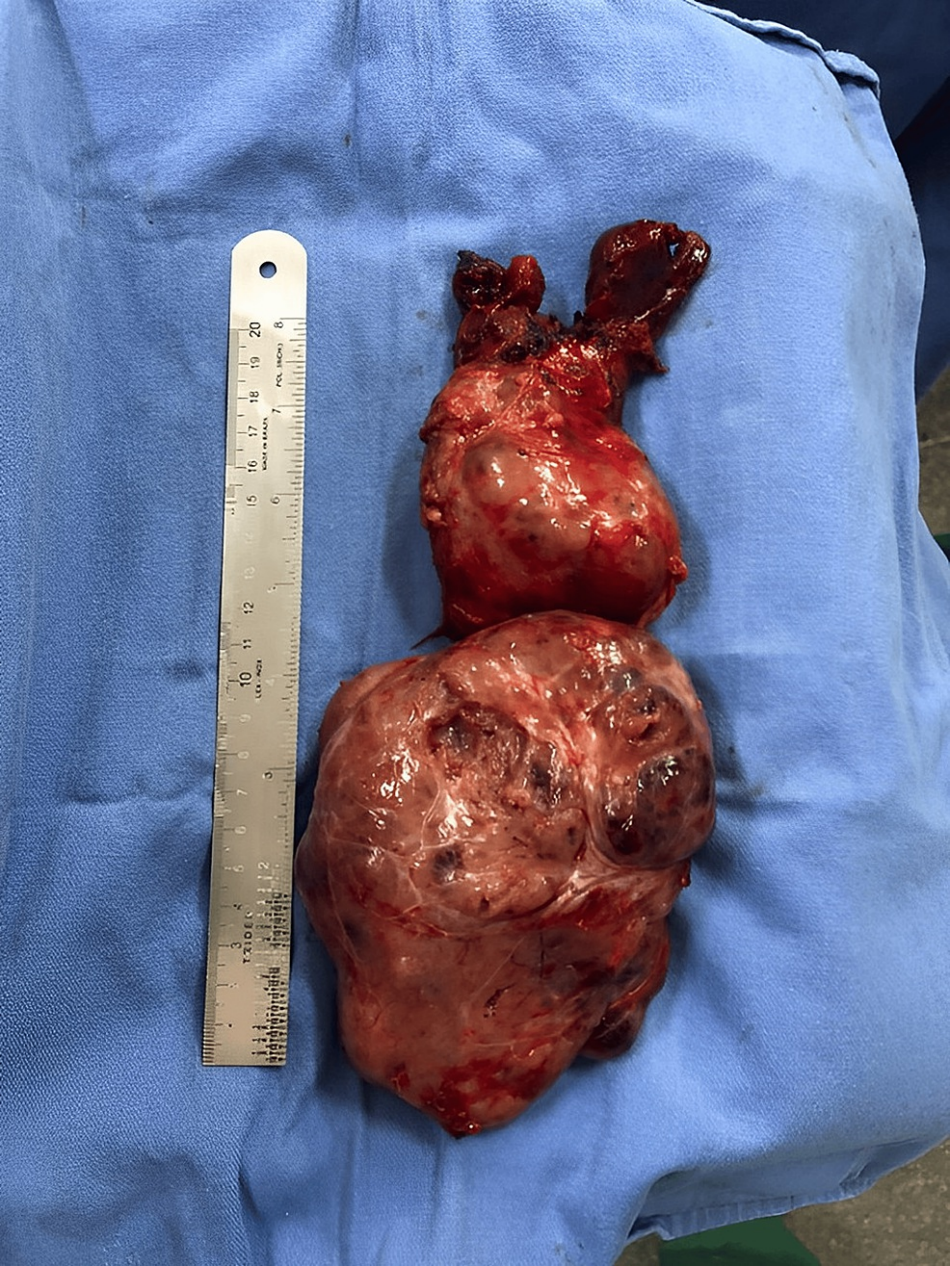
Giant substernal goiter measuring 22.5 cm in its largest extension.

The incision was amplified with a partial median sternotomy and two thyroid foci were identified. The first one, spanned from the thyroid cartilage to the sternal angle of Louis, the second one, spanned from this point to a point 3 cm above the xiphoid process. A total thyroidectomy with preservation of the parathyroid glands was performed without intraoperative complications and the partial median sternotomy was closed with stainless steel wires (Figure 2). The gross specimen sent to histopathology consisted of two conjugated thyroid lobules, the greater lobule measured 13.0 x 6.0 x 5.2 cm while the lesser one measured 9.5 x 6.0 x 5.2 cm (Figure 3). The total specimen weighed 595 grams and measured 22.5 cm in its largest extension. The histopathologic report showed multinodular goiter with cystic degeneration and dystrophic calcifications.

The patient was discharged four days after the surgery was performed with a clean surgical wound and no complaints. She was scheduled for follow-up with the thoracic surgery and endocrinology teams.

## Discussion

The management of goiter is somewhat controversial; while surgery is the treatment of choice for symptomatic patients, the treatment of asymptomatic substernal goiter has been a topic of debate. Some physicians defend an expectant approach with annual exams and avoidance of iodine-containing products. Others, propose surgical treatment for asymptomatic patients due to concern that the thyroid tissue may compress mediastinal structures. No longitudinal studies have been made assessing the potential growth of substernal goiter and, until then, this will remain a topic of controversy [2]. Another reason to treat asymptomatic patients is due to fear of undiagnosed thyroid cancer within the mass. A retrospective study conducted with 212 substernal goiters showed that the incidence of malignancy on histology was 16% [3]. Furthermore, a case-control study performed in Istanbul compared the frequency of cancer in patients treated for retrosternal goiter versus nontoxic multinodular goiter; it concluded that there was no statistically significant difference in the frequency or location of cancer between the groups. However, ultrasound was shown to not be reliable in detecting cancer in the thoracic region of retrosternal goiters, making follow-up difficult [4].

When indicated, the treatment of substernal goiter should be performed at a tertiary center with experience in the procedure to ensure optimal results. Most surgeries can be performed via a transcervical approach with a very low rate of complications. In a retrospective study with 1767 cases of substernal goiter, 99% of patients were treated with a transcervical approach. The most common complications were postoperative bleeding (0.5%), unilateral recurrent laryngeal nerve palsy (1.3%), bilateral nerve palsy (0.6%), transient hypoparathyroidism (14%) and permanent hypoparathyroidism (4.1%). While sternotomy and thoracotomy are reserved for selected cases, the lack of these options may be dangerous when attempting the cervical approach [5]. The case report presented illustrates how the option to perform a partial sternotomy may prove to be useful when the cervical approach is insufficient to fully resect the mass.

## Conclusions

In conclusion, substernal goiter is an uncommon form of goiter which predominantly affects elderly women. It is defined by the expansion of thyroid tissue into the anterior mediastinum, being an important differential diagnosis for anterior mediastinal masses. This condition may present with hypothyroidism, hyperthyroidism or be an incidental finding in an imaging exam. There is some controversy regarding when to treat substernal goiter; however, symptomatic cases should be promptly managed through surgical intervention. A transcervical approach can be used for the majority of cases and provides an adequate window to resect the mediastinal mass. Nonetheless, surgery should be performed at specialized centers as some of the cases may require expanding the initial incision with a sternotomy or thoracotomy. This case illustrates how a partial sternotomy can be used to extend the incision during a transcervical approach and facilitate resection while avoiding complications which, albeit rare, can be disastrous.
